# Strong predictive value of mannose-binding lectin levels for cardiovascular risk of hemodialysis patients

**DOI:** 10.1186/s12967-016-0995-5

**Published:** 2016-08-05

**Authors:** Felix Poppelaars, Mariana Gaya da Costa, Stefan P. Berger, Solmaz Assa, Anita H. Meter-Arkema, Mohamed R. Daha, Willem J. van Son, Casper F. M. Franssen, Marc A. J. Seelen

**Affiliations:** 1 Department of Internal Medicine, Division of Nephrology, University Medical Center Groningen, University of Groningen, Groningen, The Netherlands; 2 Department of Cardiology, University Medical Center Groningen, University of Groningen, Groningen, The Netherlands; 3 Department of Nephrology, Leiden University Medical Center, University of Leiden, Leiden, The Netherlands

**Keywords:** Complement, MBL, Cardiovascular, Risk, Hemodialysis

## Abstract

**Background:**

Hemodialysis patients have higher rates of cardiovascular morbidity and mortality compared to the general population. Mannose-binding lectin (MBL) plays an important role in the development of cardiovascular disease. In addition, hemodialysis alters MBL concentration and functional activity. The present study determines the predictive value of MBL levels for future cardiac events (C-event), cardiovascular events (CV-event) and all-cause mortality in HD patients.

**Methods:**

We conducted a prospective study of 107 patients on maintenance hemodialysis. Plasma MBL, properdin, C3d and sC5b-9 was measured before and after one dialysis session. The association with future C-events, CV-events, and all-cause mortality was evaluated using Cox regression models.

**Results:**

During median follow-up of 27 months, 36 participants developed 21 C-events and 36 CV-events, whereas 37 patients died. The incidence of C-events and CV-events was significantly higher in patients with low MBL levels (<319 ng/mL, lower quartile). In fully adjusted models, low MBL level was independently associated with increased CV-events (hazard ratio 3.98; 95 % CI 1.88–8.24; P < 0.001) and C-events (hazard ratio 3.96; 95 % CI 1.49–10.54; P = 0.006). No association was found between low MBL levels and all-cause mortality. Furthermore, MBL substantially improved risk prediction for CV-events beyond currently used clinical markers.

**Conclusions:**

Low MBL levels are associated with a higher risk for future C-events and CV-events. Therefore, MBL levels may help to identify hemodialysis patients who are at risk to develop cardiovascular disease.

**Electronic supplementary material:**

The online version of this article (doi:10.1186/s12967-016-0995-5) contains supplementary material, which is available to authorized users.

## Background

Hemodialysis (HD) is a life-saving therapy for patients with end-stage renal disease. Despite modern technology and medicine, dialysis patients still have a poor prognosis [1]. Cardiovascular disease is the leading cause of both morbidity and mortality in patients receiving HD [2]. However, the mechanism behind cardiovascular disease in these patients might not be similar to those operating in the general population [3]. The clinical need for better predictors of cardiovascular disease in dialysis patients is on-going, since traditional risk factors are insufficient to explain their extensive cardiovascular risk. Dialysis patients have accelerated atherosclerosis. The chronic inflammatory state triggered by HD is thought to be partly responsible for the accelerated atherosclerosis in HD patients [4].

The complement system is an essential part of the innate immune system, but also plays a pivotal role in the pathogenesis of a variety of diseases. It consists of three activation pathways; the classical pathway, the lectin pathway (LP) and the alternative pathway [5]. The main initiator of the LP is mannose-binding lectin (MBL), which can interact with different carbohydrate ligands found on pathogens, and on stressed or apoptotic cells [6, 7]. In the general population, there is a wide variation in plasma MBL levels, caused by genetic polymorphisms of the *mbl2 gene* [8]. MBL is also an acute phase protein and, therefore, levels can increase by two- to threefold during inflammation [9].

Considering the important role of innate immunity and its potent component MBL in inflammation, much attention has been paid to its role in the development of cardiovascular disease [10]. In clinical studies MBL has been associated with cardiovascular risk [11]. Low MBL levels as well as MBL deficiency-associated genotypes have been reported to increase cardiovascular risk in healthy individuals, independently of traditional risk factors [12, 13]. Furthermore, higher MBL markedly decreased the risk for cardiac events in individuals with diabetes, hypercholesterolemia, or chronic inflammation. Remarkably, there was no difference in the cardiovascular risk between diabetic and nondiabetic patients with MBL titers above 1000 ng/mL [12]. Moreover, experimental studies have shown that MBL is involved in the pathophysiology of atherosclerosis [14–16]. However, the relationship between MBL and disease is complex and MBL can be detrimental or beneficial depending on different genetic and environmental factors.

In HD patients, concentration and functional activity of MBL are altered compared to healthy controls [17]. MBL has also been shown to bind to the dialysis membrane during HD [18]. In addition, dialysis patients have significantly reduced levels of functional (high order oligomers) MBL, while non-functional (low-order oligomers) MBL levels are increased [19]. The basic structural unit of MBL can oligomerizes to form dimers up to hexamers. Functional MBL consists of higher order multimers (tetramers, pentamers and hexamers) [8, 20, 21]. Only high order oligomers have the ability to bind carbohydrates and activate the LP. In contrast, monomers and low order oligomers (dimers and trimers) cannot efficiently bind carbohydrates and can therefore not activate the complement system. How dialysis impacts the oligomerisation of MBL remains unknown. Nevertheless, reports about the effects of MBL levels in HD patients on clinical outcome, such as cardiovascular events, are lacking.

We hypothesized that MBL levels adversely affect cardiovascular risk in HD patients. Therefore, this study aimed to determine the predictive value of MBL levels for cardiovascular events and all-cause mortality in HD patients.

## Methods

### Study population and design

A prospective study of 45 months was conducted in a cohort of 109 hemodialyses (HD) patients, recruited at the Dialysis Center Groningen and the University Medical Center Groningen. The protocol has been described previously [22]. In brief, patients were eligible for entry when they had been on HD therapy for more than 3 months. Patients with severe heart failure (NYHA class IV) were excluded. In addition, two patients were excluded due to lack of plasma samples.

### Dialysis settings

Patients were on a three-times weekly dialysis schedule using a low-flux polysulfone hollow-fiber dialyzer (F8; Fresenius Care, Bad Homburg, Germany). The temperature of the dialysate was 36.0 or 36.5 °C. Ultrafiltration rate was constant and blood and dialysate flows were 250–350 and 500 mL/min, respectively. Blood samples were obtained at the start and end of a regular 4-h HD session.

### Clinical and laboratory measurements

Relevant patient characteristics were extracted from patient records. More details of the cohort has been published previously [22]. Clinical parameters were measured before and after dialysis. Ultrafiltration rate was calculated as described previously [23].

Laboratory measurements at baseline included hematocrit, HbA1c, albumin, pH, calcium and phosphate, which were measured by routine laboratory procedures. High-sensitivity C-reactive protein (hsCRP) was measured with the CRP monoassay (Siemens Healthcare Diagnostics).

### Plasma mannose-binding lectin levels

Plasma mannose-binding lectin (MBL) levels were assessed by ELISA as described previously [24, 25]. In short, 96-well ELISA plates were coated overnight with the anti-MBL 3E7 antibody (Hycult, Uden, The Netherlands). After blocking with 1 % BSA/PBS for 60 min, plasma EDTA samples were incubated in the coated wells. Next, wells were incubated with Dig-conjugated 3E7. Detection of binding of Dig-conjugated antibodies was performed using HRP-conjugated sheep anti-Dig Abs (Fab fragments, Roche, Mannheim, Germany). The plate was washed in PBS Tween-20 (0.05 %) between each step. For visualization 3,3′,5,5′-Tetramethylbenzidine (TMB) was added and the colorimetric reaction was stopped with H_2_SO_4_. Absorption was measured at 450 nm. Plasma from 50 healthy volunteers served as controls.

### Quantification of the antigenic levels of C3d, C3, Properdin and C5b-9

Complement activation product C3d and C5b-9 were determined. Additionally, properdin and C3 plasma concentrations were measured. Properdin, C3d, and sC5b-9 were measured as described earlier [24, 26, 27]. Quantitative antigenic assay for C3 was performed by the radial immunodiffusion technique with monospecific anti-sera [28]. Plasma from 35 healthy volunteers served as controls.

### Definition of endpoint

The primary end-point was the time to the first C-event and CV-event. The secondary outcome was all-cause mortality. C-event was defined as the occurrence of ischemic heart disease [unstable angina pectoris, myocardial infarction, Coronary Artery Bypass Grafting (CABG) and/or Percutaneous Coronary Intervention (PCI)], sudden cardiac death and congestive heart failure. Acute myocardial infarction was diagnosed if at least two of the three following criteria were met: clinical status, elevated heart enzymes, and EKG changes. CV-events were defined as cardiac, cerebrovascular or peripheral vascular events. Cerebrovascular events were defined as stroke, ischemic insult, or newly diagnosed >70 % stenosis of the extracranial carotid artery. Strokes and ischemic insults had to be verified by CT or MRI. Peripheral vascular disease was defined as having intermittent claudication with angiographically or sonographically proven stenosis >50 % of the major arteries of the lower limbs or ulcers caused by atherosclerotic stenosis or surgery for this disorder. Transplantation was a censoring event and the transplantation date was considered as the final follow-up date.

### Statistics

Statistical analysis was performed using SPSS version 22.0 (IBM Corporation, Chicago, IL, USA) and STATA version 14 (Statacorp, College Station, TX: StataCorp LP). Results are presented as mean ± standard deviation for normally distributed data, median [IQR] for non-normally distributed data and total number of patients with percentage [n (%)] for nominal data. Differences between groups were assessed with the student *t* test or the Mann–Whitney-U test for normally and not-normally distributed variables, respectively, and χ^2^ test for categorical variables. The Wilcoxon signed-rank test was used to compare values before and after HD. Correlations were assessed by using Spearman’s correlation. Log-rank tests were performed between groups to assess the difference in the incidence of C-events, CV-events and all-cause mortality and associations were assessed by Cox proportional hazard regression. The Harrell’s C statistic was used to assess how well a model distinguishes between patients who develop a CV-event and those who do not, while taking follow-up time into account. When outcome is binary, the Harrell’s C statistic is the equivalent of the area under the ROC curve [29]. A value of “1” indicated perfect discrimination whereas the value “0.5” indicated a performance comparable to chance. The additional value of MBL levels, post-dialysis, was determined by the integrated discrimination improvement (IDI), The IDI indicates the difference between model-based probabilities for events and non-events for the models with and without MBL [30, 31]. All statistical tests were 2-tailed with P < 0.05 regarded as significant.

## Results

### Patients characteristics

This study included a total of 107 subjects on maintenance hemodialysis (HD). There were 71 males and 36 females and their age was 62.5 ± 15.6 years. At baseline, the duration of HD therapy was 25.5 months (IQR 8.5–52.3 months). Hypertension was seen in 80 % of the subjects and diabetes in 23 %. Previous cardiovascular event (CV-event) was documented in 40 % of patients, specifically myocardial infarction (14 %), previous PCI/CABG (19 %), unstable angina pectoris (3 %), cerebrovascular events (14 %), or peripheral vascular disease (5 %). During median follow-up of 27 months, 36 participants developed 21 C-events and 36 CV-events, whereas 37 patients died. The maximum follow-up period was 45 months.

### MBL, properdin, C3d and C5b-9 levels in hemodialysis patients

**Fig. 1:**
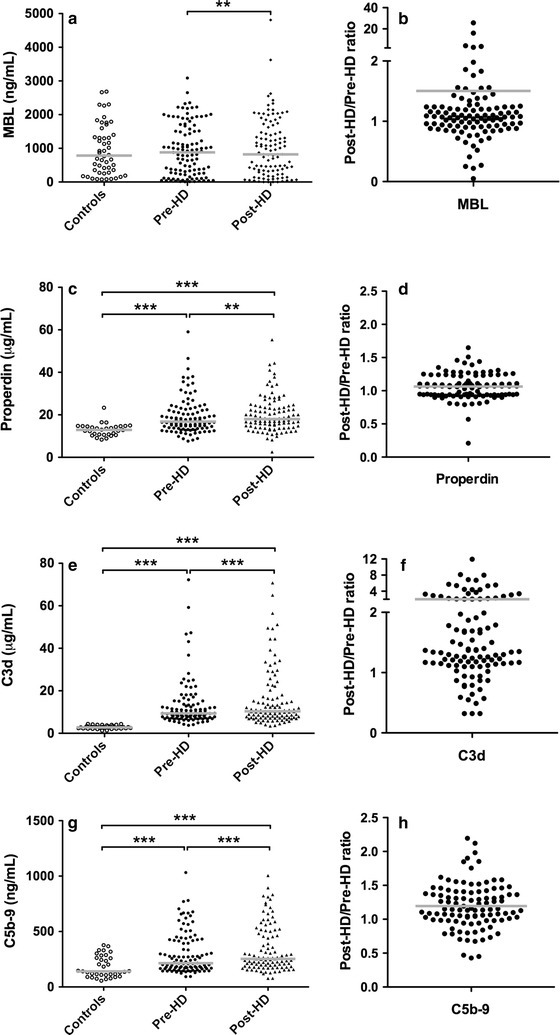
Patient plasma levels of mannose-binding lectin, properdin, C3d and C5b-9 before, after and relative change during hemodialysis. The distribution of plasma mannose-binding lectin (MBL) (**a**), properdin (**c**), C3d (**e**) and sC5b-9 (**g**) levels in healthy controls and hemodialysis (HD) patients at the start and end of the HD session.* Horizontal lines* indicate the median. The ratio for MBL (**b**), properdin (**d**), C3d (**f**) and sC5b-9 (**h**) was calculated per patient by dividing the pre-HD level by the post-HD level.* Horizontal lines* indicate the mean. A post/pre-HD ratio higher than 1, indicates an increase in concentration during HD. Differences between healthy controls and HD patients were assessed with the Mann–Whitney-U test. The Wilcoxon signed-rank test was used to compare values before and after HD. (*P < 0.05, **P < 0.01, ***P < 0.001)

Mannose-binding lectin (MBL) levels were determined before starting and at the end of the HD session. Paired analysis of MBL levels revealed a modest, but significant increase in plasma concentration after HD (Fig. 1a). This is also shown by the post/pre-HD ratio of the MBL levels (Fig. 1b). However, MBL levels were not significantly higher in HD patients compared to healthy controls (Table 1).

**Table 1:** Univariate analysis of MBL, properdin, C3, C3d and C5b-9

Plasma concentration	At start hemodialysis	At end hemodialysis	P-value^a^	Controls	P-value^b^
MBL	879 (255–1572)	821 (319–1477)	*0.005*	784 (277–1449)	0.9
Properdin	16.8 (13.6–22.4)	18.0 (14.2–23.8)	*0.01*	13.0 (10.8–14.7)	<*0.0001*
C3d	7.3 (5.6–10.1)	10.3 (7.4–16.9)	<*0.0001*	2.7 (2.3–3.4)	<*0.0001*
C5b-9	214 (166–419)	253 (187–487)	<*0.0001*	141 (107–262)	<*0.0001*

Plasma concentration	No CV-event	CV-event	P-value^c^		
MBL	1074 (428–1722)	464 (111–1102)	*0.006*		
Properdin	18.6 (14.0–24.0)	17.3 (15.1–23.4)	0.8		
C3d	10.3 (7.3–16.7)	10.8 (7.4–17.2)	0.8		
C3d/C3	8.2 (6.3–13.7)	8.6 (6.0–15.6)	0.5		
C3	1.28 (1.08–1.53)	1.32 (1.14–1.55)	0.6		
C5b-9	249 (182–513)	266 (194–473)	0.8		

% CV event-free survival	Low level (%)	High level (%)	P-value^d^		
MBL^e^	42.30	74.10	*0.003*		
MBL^f^	48.50	74.30	*0.02*		
Properdin^e^	65.40	74.10	0.6		
C3d^g^	67.50	66.70	0.8		
C3d/C3^g^	67.50	66.70	0.9		
C3^g^	68.80	63.00	0.7		
C5b-9^g^	67.50	69.20	0.4		

Values are expressed as median (interquartile range). Increasedlevels of MBL, properdin, C3d and C5b-9 were found at the end of hemodialysis compared with at the start of hemodialysis and controls. MBL was significantly lower in hemodialysis patients suffering from a cardiovascular event. An association was found between MBL and the cumulative incidence of a cardiovascular event

Italic values used to show which statistical testing was significant (below 0.05)

*CV*-*event* cardiovascular event; *MBL* mannose-binding lectin

^a^ Wilcoxon signed-rank test, at start hemodialysis vs. at end hemodialysis. All P-values are two-sided

^b^ Mann–Whitney test, at end hemodialysis vs. controls. All P-values are two-sided

^c^ Mann–Whitney test. All P-values are two-sided

^d^ Log-rank test

^e^ Split by lowest 25 %

^f^ Split by 400 ng/mL

^g^ Split by highest 25 %

To determine the contribution of the alternative pathway and complement activation, properdin, C3d and C5b-9 were analyzed. Properdin (Fig. 1c, d), C3d (Fig. 1e, f) and C5b-9 (Fig. 1g–h) levels were significantly higher at the end of the HD session compared to the start, demonstrating hemodialysis-induced complement activation. In accordance, spearman’s correlation revealed that C5b-9 levels correlated significantly with C3d levels (C5b-9/C3d, r = 0.367, P < 0.001), indicating that central complement activation is correlated with terminal complement activation. Furthermore, properdin, C3d, and C5b-9 levels were significantly higher in HD patients than healthy controls (Table 1). However, properdin, C3d and C5b-9 levels were not correlated with MBL levels in HD patients (Properdin/MBL, r = 0.049, P = 0.6; C3d/MBL, r = −0.031, P = 0.7; C5b-9/MBL, r = −0.051, P = 0.6).

### Hemodialysis patients with versus hemodialysis patients without cardiovascular events

To assess the effect of the complement system on cardiovascular risk, post-HD levels of patients who developed a CV-event during follow-up were compared to patients who did not. MBL levels were significantly lower in HD patients who developed a CV-event compared to HD patients without a CV-event (Table 1). Although not significant, subjects with CV-events tended to have lower levels of properdin and higher C3d/C3-ratio’s and C5b-9 levels than those without. We also found significant differences in ultrafiltration volume, diabetes (incidence, as primary renal disease and in HbA1c), cardiovascular history, hsCRP and medication between subjects with and without CV-events (Additional file 1: Table S1).

**Fig. 2:**
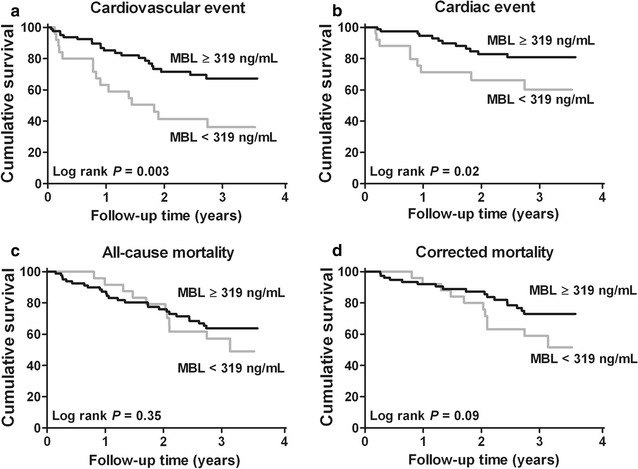
Kaplan-Meier curves for cardiovascular event, cardiac-event and all-cause mortality of hemodialysis patients with low or high mannose-binding lectin plasma levels. Cumulative event-free survival for cardiovascular events (**a**), cardiac-events (**b**), all-cause mortality (**c**) and correct mortality (**d**) among hemodialysis patient (HD) with low and high mannose-binding lectin (MBL) levels. Corrected mortality included cardiovascular, infectious and unknown mortality, while mortality for other reasons and discontinuation of dialysis therapy were excluded, Log-rank test was used to compare the incidence of cardiovascular events, cardiac-events and all-cause mortality between the groups

For further analysis, we divided our study population into groups of low complement levels and high complement levels (Table 1). Since MBL levels were significantly lower in subjects with a CV-event, the 25th percentile was used as cut-off. This was also done for properdin levels. Since C3d, C3, and C5b-9 levels were higher in subjects with a CV-event, the 75th percentile was used as cut-off. Univariate regression analysis showed a significant association between lower MBL levels and cardiovascular events. Of subjects with MBL levels below the 25th percentile, 57.7 % developed a CV-event compared to 25.9 % of the subjects with MBL levels above the 25th percentile. Kaplan–Meier analysis revealed an increased incidence of both CV-events and C-event in HD patients with low MBL levels, but not all-cause mortality (Fig. 2). However, after exclusion of death by discontinuation of dialysis therapy (n = 7) and other causes (n = 2), a trend was seen for an increased mortality rate in HD patients with low MBL levels. Conversely, properdin, C3d, C3, C3d/C3-ratio and C5b-9 levels were not associated with C-events, CV-events and all-cause mortality in HD patients.

For additional analysis, patients were divided into two groups according to MBL levels using the cut-off of 400 ng/ml. This has earlier been shown to be closely related to MBL variant alleles, whereas MBL levels above 400 ng/ml were related to MBL wild type [32]. A similar significant difference in the incidence of CV-events was observed using this cutoff. In our HD population, 30.8 % had MBL levels below 400 ng/ml and 69.2 % above, which is comparable to the frequency of variant alleles determined by others [33].

### Cardiovascular risk according to MBL levels

We set out to further investigate the predictive value of plasma MBL levels for cardiovascular risk of HD patients. There was no significant difference in baseline characteristics between the groups, according to MBL levels (Table 2). Furthermore, MBL levels correlated weakly with age (r = −0.26, P = 0.007), post-dialysis systolic blood pressure (r = −0.24, P = 0.02) but not with high-sensitivity C-reactive protein (hsCRP), body mass index (BMI), HbA1c and albumin (Table 2).

**Table 2:** Baseline characteristics of hemodialysis patients presented as groups according to MBL levels

MBL range (ng/mL)	Patients			P^*^ < 0.001	*R*	P^*#*^
	All (n = 107)	MBL low 319 < ng/mL (n = 26)	MBL high 319 ≥ ng/mL (n = 81)			
	821[319–1477]	98[33–146]	1290[671–1848]			
*Demographics*						
Age, years	62.5 ± 15.6	65.3 ± 12.1	61.56 ± 16.6	0.3	–0.26	*0.007*
Male gender, n (%)	71 (66)	17 (65)	54 (67)	1.0		
Current diabetes, n (%)	25 (24)	9 (35)	16 (20)	0.2		
Hypertension, n (%)	85 (84)	22 (88)	63 (83)	0.8		
Cardiovascular history, n (%)	26 (25)	9 (35)	15 (19)	0.1		
BMI, kg/m^2^	25.8 ± 4.4	27.0 ± 4.5	25.4 ± 4.4	0.1	–0.03	0.8
*Hemodialysis*						
Dialysis vintage, months	25.5 [8.5–52.3]	18.2 [7.0–47.7]	32.8 [9.1–53.3]	0.2	–0.01	0.9
*Primary renal disease, n (%)*						
Hypertension	18 (17)	4 (15)	14 (17)	1.0		
Diabetes	14 (13)	5 (19)	9 (11)	0.3		
ADPKD	13 (12)	3 (12)	10 (12)	1.0		
FSGS	9 (8)	4 (15)	5 (6)	0.2		
IgA nephropathy	4 (4)	0 (0)	4 (5)	0.6		
Chronic pyelonephritis	3 (3)	0 (0)	3 (4)	1.0		
Glomerulonephritis	13 (12)	2 (8)	11 (14)	0.7		
Other diagnoses	16 (16)	6 (23)	10 (12)	0.2		
Unknown	17 (16)	2 (8)	15 (19)	0.2		
Ultrafiltration volume, L	2.55 ± 0.78	2.54 ± 0.82	2.56 ± 0.78	0.9	–0.01	0.9
Ultrafiltration rate, ml/kg/h	8.56 ± 2.63	7.81 ± 2.39	8.80 ± 2.67	0.1	0.04	0.7
*Systolic blood pressure*						
Predialysis, mmHg	140.4 ± 25.1	144.7 ± 26.4	139.1 ± 24.7	0.3	–0.17	0.08
Postdialysis, mmHg	131.8 ± 25.6	136 ± 24.3	130.4 ± 26.0	0.4	–0.24	*0.02*
*Heart rate*						
Predialysis, bpm	73 [63–82]	71 [62–82]	74 [64–82]	0.3	0.11	0.3
Postdialysis, bpm	79 [69–87]	75 [65–86]	79 [69–88]	0.4	0.13	0.2
Kidney transplant, n (%)	21 (20)	4 (15)	17 (21)	0.8		
*Laboratory measurements*						
Hematocrit, %	34.9 ± 3.8	34.5 ± 4.1	35.0 ± 3.7	0.6	0.04	0.7
HbA1c, mmol/mol	5.68 ± 0.98	5.80 ± 0.97	5.63 ± 0.98	0.5	–0.15	0.2
Albumin, g/L	39 [37–42]	39 [37–42]	39 [37–42]	0.9	0.01	0.9
pH	7.37 [7.34–7.39]	7.37 [7.32–7.39]	7.37 [7.34–7.39]	0.7	0.05	0.6
Calcium, mmol/L	2.31 ± 0.16	2.31 ± 0.15	2.32 ± 0.16	0.9	0.03	0.7
Phosphate, mmol/L	1.67 ± 0.53	1.82 ± 0.47	1.65 ± 0.54	0.2	–0.00	0.9
hsCRP, mg/L	6.7 [2.8–10.9]	6.1 [1.4–12.0]	6.7 [3.0–10.9]	0.7	0.10	0.3
*Medication*						
Aspirin, n (%)	57 (54)	11 (42)	46 (64)	0.3		
Calcium channel blockers, n (%)	14 (13)	3 (12)	11 (14)	1.0		
β-Blocker, n (%)	61 (57)	18 (69)	43 (53)	0.2		
ACE inhibitor, n (%)	10 (10)	3 (12)	7 (9)	0.7		
AT2-receptor antagonists, n (%)	14 (13)	2 (8)	12 (15)	0.5		
Statin, n (%)	20 (19)	5 (19)	15 (19)	1.0		
Diuretics, n (%)	8 (8)	3 (12)	5 (6)	0.4		

Italic values used to show which statistical testing was significant (below 0.05)

*BMI* body mass index; *ADPKD* autosomal dominant polycystic kidney disease; *FSGS* focal segmental glomerulosclerosis; *HbA1c* Hemoglobin A1c; *pH* potential hydrogen; *hsCRP* high sensitive C-reactive protein; *ACE inhibitor* angiotensin-converting-enzyme inhibitor; *AT2* receptor antagonists, Angiotensin II receptor antagonists

P^*^ indicates P-value for the difference in baseline characteristics between the MBL groups, tested by Student’s t-Test or Mann–Whitney U test for continuous variables and with χ^2^ test for categorical variables; *R* indicates Spearman correlation coefficient between MBL levels and the baseline characteristic; ^#^ P indicates the corresponding P-value

Data are presented as mean ± SD or median [IQR]

Multivariate analysis was performed to adjustment for potential confounders, including age and gender, characteristics of HD (ultrafiltration volume and dialysis vintage), risk factors (cardiovascular history, diabetes, and systolic blood pressure) and inflammation (hsCRP) (Table 3). In the crude model, Low MBL levels were associated with a hazard ratio of 2.64 (95 % CI 1.36–5.13; P = 0.004) for a CV-event and 2.60 (95 % CI 1.10–6.18; P = 0.03) for a C-event. After adjustment, the hazard ratio for future CV-event was 3.98 (95 %CI 1.88–8.24; P < 0.001) or 3.96 (95 % CI 1.49–10.54; P = 0.006) for C-event in HD patients with low MBL levels. In the unadjusted and adjusted models, these associations were also significant for plasma MBL levels as a continuous variable. Subsequently, analysis of the type of CV-events revealed that HD patients with low MBL levels are more prone to develop CV-events related to atherosclerosis, but not congestive heart failure (Table 4). In addition, the percentage of cardiovascular deaths was 15 % in the low MBL group compared to 9 % in the high MBL group (Table 4).

**Table 3:** Associations of MBL levels with cardiovascular events and cardiac events in 107 chronic hemodialysis patients

	Low MBL	Log MBL continuous	*P*	HR (per SD)	95 % CI	P
	HR	95 % CI				
*Cardiovascular events*						
Model 1	2.64	1.36–5.13	*0.004*	0.64	0.46–0.90	*0.01*
Model 2	2.75	1.39–5.44	*0.004*	0.61	0.43–0.88	*0.008*
Model 3	2.94	1.45–5.94	*0.003*	0.61	0.42–0.89	*0.01*
Model 4	3.55	1.70–7.40	*0.001*	0.58	0.40–0.84	*0.004*
Model 5	3.98	1.88–8.42	<*0.001*	0.56	0.38–0.81	*0.002*
*Cardiac events*						
Model 1	2.60	1.10–6.18	*0.03*	0.71	0.46–1.10	0.1
Model 2	2.49	1.04–5.96	*0.04*	0.73	0.46–1.16	0.2
Model 3	2.65	1.08–6.55	*0.03*	0.74	0.47–1.18	0.2
Model 4	3.82	1.48–9.87	*0.006*	0.62	0.38–1.01	0.06
Model 5	3.96	1.49–10.54	*0.006*	0.59	0.35–0.98	*0.04*

Model 1: crude

Model 2: adjusted for age and gender

Model 3: adjusted for model 2 plus ultrafiltration volume and dialysis vintage

Model 4: adjusted for model 3 plus cardiovascular history, diabetes and post-HD systolic blood pressure

Model 5: adjusted for model 4 plus hsCRP

Data are presented as hazard ratio (HR) plus 95 % confidence interval (CI) according to the cut-off of MBL and per standard deviation (SD) MBL decrease

Italic values used to show which statistical testing was significant (below 0.05)

*MBL* mannose-binding lectin; *HD* hemodialysis; *hsCRP* high sensitive C-reactive protein

**Table 4:** Type of cardiovascular events and cause of death in hemodialysis patients

Cardiovascular events						
	Acute coronary syndrome	CABG/PCI	Congestive heart failure	Sudden death	CVA	Peripheral vascular disease
Low MBL levels	4 (15 %)	5 (19 %)	0 (0 %)	2 (8 %)	2 (8 %)	4 (15 %)
High MBL levels	4 (5 %)	5 (6 %)	3 (4 %)	3 (4 %)	2 (2 %)	8 (10 %)

Mortality					
	Cardiovascular	Infection	Stopping dialysis therapy	Others	Unknown
Low MBL levels	4 (15 %)	1 (4 %)	0 (0 %)	0 (0 %)	6 (23 %)
High MBL levels	7 (9 %)	1 (1 %)	7 (9 %)	2 (2 %)	9 (11 %)

Data are given as an absolute number of cardiovascular events or cause of death and as a percentage (%) of the total number of HD patients in each MBL group

*CABG* coronary artery bypass graft; *PCI* percutaneous coronary intervention; *CVA* cerebrovascular accident

### Predictive Value of MBL

The additional value of MBL for risk prediction of cardiovascular events was assessed (Table 5). The Harrell’s C statistic was used to investigate the capability of each model to predict cardiovascular events and to compare the additional value of MBL levels in the different models. Plasma MBL alone had a Harrell’s C of 0.64 (0.54–0.75). Furthermore, Harrell’s C in Table 5 show that the more variables we adjusted for, the better the model predicted cardiovascular events. The models containing MBL improved significantly according to the integrated discrimination improvement index (IDI). Even in the fully adjusted models, the IDI value was >2 %, indicating that MBL substantially improved risk prediction for cardiovascular events beyond currently used clinical markers.

**Table 5:** Additive value of plasma MBL for the prediction of cardiovascular events in hemodialysis patients

	Harrell’s C (95 % CI)		Change (95 % CI)^a^	IDI (%)	P
	Without MBL	With MBL			
Model 1	0.56 (0.46–0.66)	0.64 (0.53–0.76)	0.085 (0.072–0.098)	5.93	0.01
Model 2	0.64 (0.55–0.73)	0.67 (0.57–0.77)	0.033 (0.028–0.038)	5.35	0.01
Model 3	0.71 (0.63–0.80)	0.74 (0.65–0.83)	0.027 (0.026–0.028)	6.05	0.01
Model 4	0.73 (0.64–0.82)	0.76 (0.68–0.85)	0.033 (0.033–0.033)	6.92	0.01

Data are presented as Harrell’s concordance statistic (Harrell’s C) with 95 % confidence interval (CI) and integrated discrimination improvement (IDI) with P-value (P)

Model 1: age and gender

Model 2: age, gender, ultrafiltration volume and dialysis vintage

Model 3: age, gender, ultrafiltration volume and dialysis vintage, history of CVD, DM and post-HD systolic blood pressure

Model 4: age, gender, ultrafiltration volume and dialysis vintage, history of CVD, DM and post-HD systolic blood pressure and hsCRP

*MBL* mannose-binding lectin; *CVD* cardiovascular diseases; *DM* Diabetes Mellitus; *HD* hemodialysis; *hsCRP* high sensitive C-reactive protein

^a^Change in C-statistics compared to model without post-hemodialysis MBL levels

## Discussion

We found that lower plasma mannose-binding lectin (MBL) levels are associated with a higher incidence of cardiac (C-event) and cardiovascular events (CV-event) in hemodialysis (HD) patients. In both unadjusted and adjusted models, these associations were observed after a maximum follow-up of 45 months and were independent of established risk factors. Extending these findings, the higher cardiovascular risk for HD patients with low MBL levels seems to be attributed to CV-events linked to atherosclerosis. No significant association was found between MBL levels and all-cause mortality, but a trend was visible for corrected mortality. For the first time, evidence is provided that MBL levels are a potent predictor of cardiovascular risk in patients on maintenance HD. Even in fully adjusted models, MBL substantially improved risk prediction for cardiovascular events beyond currently used clinical markers. These results suggest that MBL has a considerable influence on the pathophysiology of CV-events in HD patients and that low levels of MBL are unfavorable for these patients.

Cardiovascular morbidity and mortality in HD patients is excessively high, with rates that are 10- to 20-fold greater than in the general population [2]. In HD patients, traditional risk factors for cardiovascular disease are often found to be related to outcome in an opposite direction, which has been referred to as “reverse epidemiology” [3]. To improve risk stratification and our understanding of the causes of cardiovascular disease in these patients, the emphasis has been placed on finding better predictors of cardiovascular morbidity and mortality. The association between MBL and CV-events has previously been reported in both the healthy population [12] and in diseases such as diabetes [34] and rheumatoid arthritis [35]. Both *mbl2* genotype and MBL levels have been associated with increased risk for CV-event. However, the role of MBL in cardiovascular disease cannot be unequivocally defined, since MBL can be either detrimental or beneficial [10]. In our study, low MBL levels were associated with future CV-events, suggesting a beneficial role for MBL in HD.

Circulating MBL levels are largely determined by the *mbl2* gene and levels vary greatly from person to person due to frequently occurring polymorphisms [8]. The incidence of MBL deficiency varies among populations [21]. Additionally, MBL levels are influenced by other factors such as age, sex, and lifestyle. In mice, MBL levels and functionality are different between genders, however, these findings have not been confirmed in humans [36, 37]. Others have shown that MBL levels decline with age [37, 38]. Moreover, lifestyle factors can also impact MBL. Fasting and dietary restrictions reduce circulating levels of MBL as well as mRNA expression in liver [39], However, after adjustment MBL for these confounders levels remained associated with cardiovascular events, indicating a direct and independent effect of MBL on cardiovascular risk. This study revealed that MBL levels are the same in HD patients and healthy controls. The mean MBL levels of 955 ng/mL in our healthy controls are comparable to the levels described previously [10]. There have been several reports about MBL levels in HD patients. Similar to us, Ishii et al. and found no difference in plasma concentration of MBL between HD patients and healthy controls [40], while other studies have provided opposite findings [17, 41]. These paradoxical results about MBL levels in HD patients are explained by differences in genetic background, race, primary renal disease and percentage of diabetic subjects of the HD population. Lastly, ELISA techniques used to determine MBL have to be taken into account [19]. Satomura et al. revealed that patients undergoing HD have significantly reduced levels of high order oligomers (functional) MBL, while the same patients have significantly increased levels of low-order oligomers (non-functional) MBL [41]. In a Dutch cohort of renal transplant recipients, MBL levels similar to our study were found in samples obtained prior to transplantation [25]. Using the same MBL ELISA setup, they also concluded that MBL levels in HD patients are identical to healthy controls. In addition, we revealed that during HD, plasma MBL levels increase significantly. Although we are the first to show changes in MBL levels during an HD session, it has previously been shown that MBL levels of HD patients were significantly higher after 6 and 12 months than at the start of HD therapy [17]. The increase in MBL concentration is, therefore, unlikely to be a cause of the ultrafiltration during dialysis.

Few studies evaluated the effect of MBL levels in HD patient on clinical outcome. Satomura et al. showed that HD patients with low MBL levels had a significantly higher all-cause mortality than patients with high MBL levels [42]. In contrast, we found no differences in all-cause mortality. A possible explanation could be the difference in the percentage of cardiovascular mortality. In their study, the majority of deaths (67 %) were cardiovascular; whereas in our study, the percentage of cardiovascular mortality was much lower (30 %). Non-cardiovascular mortality accounted for another 30 percent in our study and the cause of death for the remaining 40 percent was unknown. However, the results of Satomura et al. are in line with our finding that low MBL levels are detrimental in HD patients. In addition, in our study the percentage of cardiovascular deaths was higher in low MBL group. Recently, it was demonstrated that higher levels of C3 at baseline are associated with an increased risk of CV-events [43]. In accordance, we found higher levels of C3 in HD patients who developed a CV-event compared to HD patients without a CV-event. However, this was not significant, but this is most likely due to the smaller sample size of our study.

It has become clear that MBL is associated with cardiovascular disease. However, the relationship between MBL and disease is rather complex. Data obtained by clinical studies have been contradictory, MBL was sometimes protective and, at other times harmful. A possible explanation for this ambiguous role can be found in the different effector functions of MBL [44]. Whether these effector functions exhibit positive or negative effects in cardiovascular diseases depends on various elements, such as accompanying pathology, other risk factors, age, and sex. For instance, complement activation and thrombus formation via MBL [45] could be detrimental whereas opsonization and recognition of altered self/apoptosis by MBL would be beneficial. We postulate that in HD patients, low MBL levels increase cardiovascular risk by promoting atherosclerosis due to the defective removal of atherogenic particles. This hypothesis is supported by evidence from previous studies showing that MBL deficient subjects have worse and accelerated atherosclerosis [46, 47]. Furthermore, MBL is locally expressed during atherogenesis and negatively regulated the development of these lesions [14]. MBL is also involved in the removal of atherogenic particles and deficiency subsequently leads to accumulation of these particles [15, 16]. In patients with end-stage renal failure, low MBL levels have been linked to higher arterial stiffness [48].

Our study has limitations and strengths. Although causality of the associations found is likely, it cannot be proven since our study is prospective but observational in nature. We are aware that the proposed mechanisms described here are only speculative. Furthermore, in our HD patients genotyping of the *mbl2* gene was not performed due to the lack of DNA. MBL is an acute phase protein, so the plasma concentration increases substantially during inflammation [9]. We cannot determine if the low MBL level in HD patients was due to genetic background or an insufficient inflammatory response. However, the lack of correlation with CRP is an argument against this. Finally, the population size is relatively small, limiting our power to detect all but the strongest associations between complement and cardiovascular risk. Negative findings should be interpreted with caution due to the risk of false negative associations. On the other hand, strengths include the long follow-up and uniform single-center handling of samples along with the hard and clinically relevant endpoints (C-events and CV-events).

## Conclusion

In summary, measurement of plasma MBL level may proof to be a novel diagnostic tool and functional biomarker of cardiac and cardiovascular risk in HD patients, which may substantially improve prognostication. Intervention studies based on plasma MBL concentrations are required to clarify whether therapeutic targeting improves the cardiovascular risk of patients on maintenance HD.

## Additional file

10.1186/s12967-016-0995-5 Baseline characteristics of our study population of hemodialysis patients with and without a cardiovascular event.

## Abbreviations

ADPKD: autosomal dominant polycystic kidney disease
BMI: body mass index
BSA: bovine serum albumin
C5b-9: membrane attack complex
C-event: cardiac event
CABG: coronary artery bypass grafting
CI: confidence interval
CV-event: cardiovascular event
CVA: cerebrovascular accident
EDTA: ethylenediaminetetraacetic acid
ELISA: enzyme-linked immunosorbent assay
FSGS: focal segmental glomerulosclerosis
HbA1c: glycated hemoglobin A1c
HD: hemodialysis
HRP: horseradish peroxidase
hsCRP: high sensitive C-reactive protein
IDI: integrated discrimination improvement
IQR: interquartile range
LP: lectin pathway
MASP-1: MBL-associated serine proteases 1
MASP-2: bMBL-associated serine proteases 2
MBL: mannose-binding lectin
PCI: percutaneous coronary intervention
