# Erratum to: Strong predictive value of mannose-binding lectin levels for cardiovascular risk of hemodialysis patients

**DOI:** 10.1186/s12967-016-1004-8

**Published:** 2016-08-24

**Authors:** Felix Poppelaars, Mariana Gaya da Costa, Stefan P. Berger, Solmaz Assa, Anita H. Meter-Arkema, Mohamed R. Daha, Willem J. van Son, Casper F. M. Franssen, Marc A. J. Seelen

**Affiliations:** 1 Division of Nephrology, Department of Internal Medicine, University Medical Center Groningen, University of Groningen, Groningen, The Netherlands; 2 Department of Cardiology, University Medical Center Groningen, University of Groningen, Groningen, The Netherlands; 3 Department of Nephrology, Leiden University Medical Center, University of Leiden, Leiden, The Netherlands

## Erratum to: J Transl Med (2016) 14:236 DOI:10.1186/s12967-016-0995-5

Unfortunately, the original version of this article [1] contained errors in the main text and in Tables 2 and 3. Tables 2 and 3 were included incorrectly. The correct Tables 2 and 3 have been updated in the original article and are also included correctly in this erratum.

**Table 2:** Baseline characteristics of hemodialysis patients presented as groups according to MBL levels

	Patients			P^*^ < 0.001	R	P^#^
	All (n = 107)	MBL low 319 < ng/mL (n = 26)	MBL high 319 ≥ ng/mL (n = 81)			
MBL range (ng/mL)	821 [319–1477]	98 [33–146]	1290 [671–1848]			
*Demographics*						
Age, years	62.5 ± 15.6	65.3 ± 12.1	61.56 ± 16.6	0.3	−0.26	*0.007*
Male gender, n (%)	71 (66)	17 (65)	54 (67)	1.0		
Current diabetes, n (%)	25 (24)	9 (35)	16 (20)	0.2		
Hypertension, n (%)	85 (84)	22 (88)	63 (83)	0.8		
Cardiovascular history, n (%)	26 (25)	9 (35)	15 (19)	0.1		
BMI, kg/m^2^	25.8 ± 4.4	27.0 ± 4.5	25.4 ± 4.4	0.1	−0.03	0.8
*Hemodialysis*						
Dialysis vintage, months	25.5 [8.5–52.3]	18.2 [7.0–47.7]	32.8 [9.1–53.3]	0.2	−0.01	0.9
*Primary renal disease, n (%)*						
Hypertension	18 (17)	4 (15)	14 (17)	1.0		
Diabetes	14 (13)	5 (19)	9 (11)	0.3		
ADPKD	13 (12)	3 (12)	10 (12)	1.0		
FSGS	9 (8)	4 (15)	5 (6)	0.2		
IgA nephropathy	4 (4)	0 (0)	4 (5)	0.6		
Chronic pyelonephritis	3 (3)	0 (0)	3 (4)	1.0		
Glomerulonephritis	13 (12)	2 (8)	11 (14)	0.7		
Other diagnoses	16 (16)	6 (23)	10 (12)	0.2		
Unknown	17 (16)	2 (8)	15 (19)	0.2		
Ultrafiltration volume, L	2.55 ± 0.78	2.54 ± 0.82	2.56 ± 0.78	0.9	−0.01	0.9
Ultrafiltration rate, mL/kg/h	8.56 ± 2.63	7.81 ± 2.39	8.80 ± 2.67	0.1	0.04	0.7
*Systolic blood pressure*						
Predialysis, mmHg	140.4 ± 25.1	144.7 ± 26.4	139.1 ± 24.7	0.3	−0.17	0.08
Postdialysis, mmHg	131.8 ± 25.6	136 ± 24.3	130.4 ± 26.0	0.4	−0.24	*0.02*
*Heart rate*						
Predialysis, bpm	73 [63–82]	71 [62–82]	74 [64–82]	0.3	0.11	0.3
Postdialysis, bpm	79 [69–87]	75 [65–86]	79 [69–88]	0.4	0.13	0.2
Kidney transplant, n (%)	21 (20)	4 (15)	17 (21)	0.8		
*Laboratory measurements*						
Hematocrit, %	34.9 ± 3.8	34.5 ± 4.1	35.0 ± 3.7	0.6	0.04	0.7
HbAlc, mmol/mol	5.68 ± 0.98	5.80 ± 0.97	5.63 ± 0.98	0.5	−0.15	0.2
Albumin, g/L	39 [37–42]	39 [37–42]	39 [37–42]	0.9	0.01	0.9
pH	7.37 [7.34–7.39]	7.37 [7.32–7.39]	7.37 [7.34–7.39]	0.7	0.05	0.6
Calcium, mmol/L	2.31 ± 0.16	2.31 ± 0.15	2.32 ± 0.16	0.9	0.03	0.7
Phosphate, mmol/L	1.67 ± 0.53	1.82 ± 0.47	1.65 ± 0.54	0.2	−0.00	0.9
hsCRP, mg/L	6.7 [2.8–10.9]	6.1 [1.4–12.0]	6.7 [3.0–10.9]	0.7	0.10	0.3
*Medication*						
Aspirin, n (%)	57 (54)	11 (42)	46 (64)	0.3		
Calcium channel blockers, n (%)	14 (13)	3 (12)	11 (14)	1.0		
β-Blocker, n (%)	61 (57)	18 (69)	43 (53)	0.2		
ACE inhibitor, n (%)	10 (10)	3 (12)	7 (9)	0.7		
AT2-receptor antagonists, n (%)	14 (13)	2 (8)	12 (15)	0.5		
Statin, n (%)	20 (19)	5 (19)	15 (19)	1.0		
Diuretics, n (%)	8 (8)	3 (12)	5 (6)	0.4		

Italic values used to show which statistical testing was significant (below 0.05)

Data are presented as mean ± SD or median [IQR]

*BMI* body mass index, *ADPKD* autosomal dominant polycystic kidney disease, *FSGS* focal segmental glomerulosclerosis, *HDA1c* hemoglobin A1c, *pH* potential hydrogen, *hsCRP* high sensitive C-relative protein, *ACE* inhibitor angiotensin-converting-enzyme inhibitor, *AT2 receptor antagonists* Angiotensin II receptor antagonists

P* indicates P value for the difference in baseline characteristics between the MBL groups, tested by Student’s t test or Mann–Whitney U test for continuous variables and with χ^2^ test for categorical variables; *R* indicates Spearman correlation coefficient between MBL levels and the baseline characteristic; P^#^ indicates the corresponding P value

**Table 3:** Associations of MBL levels with cardiovascular events and cardiac events in 107 chronic hemodialysis patients

	Low MBL			Log MBL continuous		
	HR	95 % CI	*P*	HR (per SD)	95 % CI	*P*
Cardiovascular events						
Model 1	2.64	1.36–5.13	*0.004*	0.64	0.46–0.90	*0.01*
Model 2	2.75	1.39–5.44	*0.004*	0.61	0.43–0.88	*0.008*
Model 3	2.94	1.45–5.94	*0.003*	0.61	0.42–0.89	*0.01*
Model 4	3.55	1.70–7.40	*0.001*	0.58	0.40–0.84	*0.004*
Model 5	3.98	1.88–8.42	<*0.001*	0.56	0.38–0.81	*0.002*
Cardiac events						
Model 1	2.60	1.10–6.18	*0.03*	0.71	0.46–1.10	0.1
Model 2	2.49	1.04–5.96	*0.04*	0.73	0.46–1.16	0.2
Model 3	2.65	1.08–6.55	*0.03*	0.74	0.47–1.18	0.2
Model 4	3.82	1.48–9.87	*0.006*	0.62	0.38–1.01	0.06
Model 5	3.96	1.49–10.54	*0.006*	0.59	0.35–0.98	*0.04*

Model 1: crude

Model 2: adjusted for age and gender

Model 3: adjusted for model 2 plus ultrafiltration volume and dialysis vintage

Model 4: adjusted for model 3 plus cardiovascular history, diabetes and post-HD systolic blood pressure

Model 5: adjusted for model 4 plus hsCRP

Data are presented as hazard ratio (HR) plus 95 % confidence interval (CI) according to the cut-off of MBL and per standard deviation (SD) MBL increase

Italic values used to show which statistical testing was significant (below 0.05)

*MBL* mannose-binding lectin, *HD* hemodialysis, *hsCRP* high sensitive C-reactive protein

Additionally, the following section has been corrected:

However, after adjustment MBL for these confounders levels remained associated with cardiovascular events, indicating a direct and independent effect of MBL on cardiovascular risk.

Should read:

However, after adjustment for these confounders, MBL levels remained associated with cardiovascular events, indicating a direct and independent effect of MBL on cardiovascular risk.
