# Supplementary material for: Strong predictive value of mannose-binding lectin levels for cardiovascular risk of hemodialysis patients
Source: J Transl Med. 2016 Aug 5;14:236. doi: 10.1186/s12967-016-0995-5 (PMC4974702; doi:10.1186/s12967-016-0995-5)
Supplement: Supplementary file 1 — 10.1186/s12967-016-0995-5 Baseline characteristics of our study population of hemodialysis patients with and without a cardiovascular event. [file 12967_2016_995_MOESM1_ESM.docx]

| **Additional file 1: Table S1.** Baseline characteristics of our study population of hemodialysis patients with and without a cardiovascular event. | | | | | |
| --- | --- | --- | --- | --- | --- |
|  | **Patients** | | | **Univariable** | |
|  | **CV-event**  **(n=36)** | **No CV-event**  **(n=71)** | ***P* *** | **St. Beta** | ***P* ^#^** |
| MBL range (ng/mL) | 464 [111-1102] | 1074 [428-1722] | **0.006** | 0.97 | **0.003** |
| **Demographics** | | | | | |
| Age, years | 64.5±11.7 | 61.4±17.3 | 0.3 | 0.01 | 0.5 |
| Male gender, n (%) | 25 (69) | 46 (65) | 0.7 | 0.36 | 0.3 |
| Current diabetes, n (%) | 15 (42) | 10 (14) | **0.003** | 1.02 | **0.002** |
| Hypertension, n (%) | 28 (82) | 57 (85) | 0.8 | -0.11 | 0.8 |
| Cardiovascular history, n (%) | 13 (36) | 11 (16) | **0.03** | 0.82 | **0.02** |
| BMI, kg/m^2^ | 26.4±5.8 | 25.5±3.8 | 0.4 | 0.04 | 0.3 |
| **Hemodialysis** | | | | | |
| Dialysis vintage, months | 21.9 [8.5-54.5] | 29.3 [11.0-51.2] | 0.9 | -0.00 | 0.7 |
| Ultrafiltration volume, L | 2.77±0.82 | 2.44±0.80 | **0.04** | 0.55 | **0.01** |
| Ultrafiltration rate, ml/kg/h | 9.05±2.58 | 8.31±2.65 | 0.2 | 0.12 | 0.07 |
| Systolic blood pressure | | | | | |
| Predialysis, mmHg | 138.9±26.0 | 141.1±24.7 | 0.7 | -0.01 | 0.4 |
| Postdialysis, mmHg | 125.2±21.7 | 135.2±27.0 | **0.04** | -0.01 | **0.04** |
| Heart rate | | | | | |
| Predialysis, bpm | 77 [67-86] | 72 [63-80] | 0.1 | 0.02 | 0.1 |
| Postdialysis, bpm | 79 [69-88] | 79 [65-87] | 0.9 | 0.00 | 0.7 |
| **Laboratory measurements** | | | | | |
| Hematocrit, % | 34.4±3.6 | 35.1±3.9 | 0.3 | -0.05 | 0.3 |
| HbA1C, mmol/mol | 6.03±1.24 | 5.46±0.71 | **0.02** | 0.35 | **0.01** |
| Albumin, g/L | 40 [37-41] | 39 [37-42] | 0.9 | 0.02 | 0.7 |
| pH | 7.37 [7.34-7.39] | 7.36 [7.34-7.39] | 0.4 | 0.00 | 0.4 |
| Calcium, mmol/L | 2.30±0.13 | 2.31±0.17 | 0.6 | -0.64 | 0.5 |
| Phosphate, mmol/L | 1.83±0.58 | 1.63±0.49 | 0.06 | 0.61 | **0.05** |
| hsCRP, mg/L | 7.2 [3.8-18.2] | 6.1 [1.5 – 8.9] | **0.04** | 0.02 | **0.04** |
| **Medication** | | | | | |
| Aspirin, n (%) | 13 (36) | 44 (62) | **0.01** | -0.89 | **0.01** |
| Calcium channel blockers, n (%) | 4 (11) | 10 (14) | 0.8 | -0.41 | 0.4 |
| β-Blocker, n (%) | 23 (64) | 38 (54) | 0.4 | 0.47 | 0.2 |
| ACE inhibitor, n (%) | 3 (8) | 7 (10) | 1.0 | -0.22 | 0.7 |
| AT2-receptor antagonists, n (%) | 2 (6) | 12 (17) | 0.1 | -1.14 | 0.1 |
| Statin, n (%) | 5 (14) | 15 (21) | 0.4 | -0.40 | 0.4 |
| Diuretics, n (%) | 4 (11) | 4 (6) | 0.4 | 1.17 | **0.02** |
| P * indicates P-value for the difference in baseline characteristics between the patient with and without a cardiovascular-event. Differences were tested by Student's t-Test or Mann–Whitney U test for continuous variables and with χ^2^ test for categorical variables. Data are presented as mean ± SD or median [IQR]. P **^#^** indicates P-value for univariate Cox-regression for the occurrence of CV-event. Data are presented as beta coefficient with corresponding P-value.  Abbreviations: MBL, Mannose-binding lectin; CV, cardiovascular; BMI, body mass index; HbA1c, Hemoglobin A1c; pH, potential hydrogen; hsCRP, high sensitive C-reactive protein; ACE inhibitor, angiotensin-converting-enzyme inhibitor; AT2-receptor antagonists, Angiotensin II receptor antagonists. | | | | | |

**Supplementary data**
